# Affective neuroscience personality traits in opioid use disorder patients: The relationship with earlier onset of substance use, the severity of addiction, and motivational factors to quit opiate use

**DOI:** 10.1002/brb3.70050

**Published:** 2024-09-24

**Authors:** Gonca Aşut, Yasemin Hoşgören Alıcı, Selvi Ceran, Mustafa Danışman, Şafak Yalçın Şahiner

**Affiliations:** ^1^ Department of Psychiatry Başkent University Faculty of Medicine Ankara Turkey; ^2^ Department of Psychiatry Ankara Training and Research Hospital Alcohol and Drug Addiction Treatment Centre Ankara Turkey; ^3^ Department of Psychiatry Ankara University Faculty of Medicine Ankara Turkey

**Keywords:** affective personality, ANGER, early onset drug use, opioid addiction

## Abstract

**Introduction:**

This study aims to explore the relationship between affective personality traits and opioid use disorder (OUD), including factors such as motivation to quit, addiction severity, and age of onset of drug use.

**Methods:**

This study included 141 patients with opioid addiction (OAP) and 160 age‐ and sex‐matched healthy controls (HC). OAP were interviewed and diagnosed according to DSM‐5 criteria. HC were screened for past or current drug use. Participants completed sociodemographic forms and the Affective Neuroscience Personality Scale (ANPS), and the OAP group also completed the Addiction Profile Index (API).

**Results:**

SEEK, PLAY, and SADNESS were identified as different affective personality traits between OAP and HC groups. Addiction severity was positively correlated with SADNESS and ANGER, while the age of onset of drug use was correlated with ANGER. Risk factors for OA include family history of substance abuse, low education, and low PLAY scores, whereas risk factors for earlier substance use onset are childhood trauma and high ANGER scores.

**Conclusions:**

This study highlights the importance of understanding affective personality traits in OUD. These findings may deepen our understanding of the underlying mechanisms of OUD. The identification of these affective systems may have implications for the development of personalized prevention and treatment strategies.

## Graphical abstract

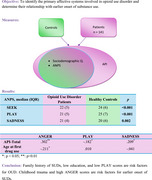

## 1 INTRODUCTION

Opioid use disorder (OUD) is a chronic brain disorder that is characterized by an overwhelming, compulsive, and compelling urge to use opiates, a preference of opiates over healthy, natural rewards, and continued use despite negative consequences (Clark et al., 2015). Risk factors for OUD include a family history of substance use disorders (SUDs), emotional instability, and a personal history of childhood trauma and untreated psychiatric disorders (Bakhshaie et al., 2019; Dydyk et al., 2023). OUD is a complex disorder that involves a combination of biological, sociological, and psychological factors. In recent years, there has been an effort to integrate psychological and neuropsychiatric perspectives to establish a cohesive theoretical understanding of opioid use disorder.

The endogenous opioidergic system is crucial for regulating mood and stress, modulating emotional and physical pain, and reward and pleasure pathways (Koob, 2020; Wright & Panksepp, 2012). Addictive drugs exploit the mesolimbic dopaminergic (MLDA) pathway, a system involved in motivation, incentive salience, and executive functions. Studies indicate that individuals struggling with distress, anxious dysphoria, and turbulent anger may be drawn to the calming effects of opiates (Majewska, 1996). It has been proposed that individuals with elevated levels of negative affectivity and emotional dysregulation, regardless of their experience with pain, may be at greater risk of developing opioid‐related problems (Bakhshaie et al., 2019). This relationship between opioid use and motivation, emotions such as anger, and negative affectivity results in the hypothesis that opioid use is a way of self‐medication for coping with these situations.

Affective neuroscience (AN) theory proposes dysregulations in subcortical affective systems as an important factor in the etiology of psychiatric disorders. In brief, AN approach has identified seven primary affective systems which arise from the periaqueductal gray matter (PAG) and expand into the limbic forebrain (Panksepp & Biven, 2012). These systems have been labeled as SEEK, CARE, LUST, and PLAY (mediating positive emotions) and FEAR, GRIEF/PANIC, and ANGER (or RAGE) (the major negative emotions) (Panksepp, 2004).

Affective neuroscience theory expanded the understanding of motivation and reward system by introducing the SEEKING system, which drives the urge to explore and anticipate rewards (Wright & Panksepp, 2012). The SEEKING system corresponds largely to the MLDA system; it seeks rewards but does not equate to pleasure. Panksepp argued that excessive SEEKING activity leads to distorted thinking, characterized by false associations between stimuli and reward anticipation. This excessive activity contributes to the paradoxical thinking seen in addiction, where drug use continues despite harmful consequences. Users may fail to recognize these false associations, increasing the risk of addiction while hindering help‐seeking behaviors (Flores Mosri, 2019, 2021; Wright & Panksepp, 2012).

In addition, some of the primary affective systems are influenced by opioidergic mechanisms. The CARE system, which involves caregiving, social bonding, and empathy, is one of these systems. Opioids enhance caregiving by producing pleasurable sensations associated with caring for another person, thereby increasing social bonding and attachment (Montag & Panksepp, 2016). The PANIC/GRIEF system, which regulates feelings of separation distress, sadness, and social loss, is also modulated by opioidergic mechanisms. Both endogenous and exogenous opioids can have a calming effect and reduce social pain (Montag & Panksepp, 2016). In this context, addiction may be understood as a dysfunctional attempt to compensate for overwhelming feelings of isolation, loss, and sadness mediated by an overactive PANIC/GRIEF system (Unterrainer et al., 2017).

Furthermore, the PLAY system, which underlies playful behavior and positive social interactions, is partly modulated by the opioidergic system. The release of endogenous opioids during social play contributes to feelings of pleasure and attachment, making play a rewarding experience. Activation of the PLAY system is associated with the release of endogenous opioids that promote feelings of happiness and social bonding (Montag & Panksepp, 2016; Panksepp, 2004). However, very little is known about the role of affective neuroscience personality traits in the etiology of OUD.

Also, negative primary emotional systems may play a role in the development of OUD. In a study, individuals with multiple drug use disorders were compared with tobacco smokers and nonsmokers; it is stated that the multiple‐drug use disorder group had higher scores of ANGER, FEAR, and SADNESS (Unterrainer et al., 2017). In another study on a sample of SUD patients, the dimensions of SADNESS and ANGER were found to play a role in depressive symptoms (Fuchshuber et al., 2018). PLAY is another affective personality trait that has a role in depressive tendencies, together with SEEK, ANGER, and FEAR (Montag et al., 2021).

To our knowledge there are no studies examining affective personality traits in patients with nonmedical OUD. Due to the lack of previous research on the relationship between primary affective systems and opioid use disorder, it is challenging to formulate a precise hypothesis regarding the potential associations between affective systems and aspects such as the motivation for opiate use, the impact of opiate use on individuals’ lives, and the severity of addiction.

This study aims to identify the primary affective systems involved in opioid use disorder and determine their relationship with earlier onset of substance use, the severity of opioid addiction, and motivational factors to quit opiate use.

## 2 MATERIAL AND METHODS

The study protocol was approved by Baskent University Institutional Review Board and Ethics Committee (Project no: KA 23/114). The sample size for this study was calculated in SPSS to provide adequate power to detect a difference in affective neuroscience personality traits between opioid use disorder patients and healthy controls. The calculation assumed a medium effect size (Cohen's *d* = .5), a significance level of.05, and a desired power of.80. Based on these parameters, the sample size calculation showed that a minimum of 138 participants per group would be required.

The study included the first 150 patients aged 18–65 who had applied for treatment for opioid use disorder at the University of Ministry of Health Ankara Training and Research Hospital Alcohol and Drug Addiction Treatment Centre (AMATEM) outpatient clinic between June 2023 and August 2023. All patients underwent a detailed psychiatric interview by an experienced psychiatrist and were diagnosed with Opioid Use Disorder according to DSM‐5 criteria. Patients who declined participation, as well as those with psychiatric or neurological conditions affecting cognitive function (i.e., any psychotic disorder, bipolar disorder, dementia), were excluded. Nine patients were not included due to incomplete forms. The aim was to obtain age‐ and sex‐matched controls without opioid use disorder. All healthy controls who agreed to participate in the study were asked about past or current drug use, and 16 healthy controls with any type of illicit drug use were excluded from the study. As a result, 141 opioid use disorder patients and 160 healthy volunteers matched for age and sex to the patients were included in the study.

All participants were informed about the study and provided an informed consent form for their participation in the study. The sociodemographic data form and the Affective Neuroscience Personality Scale (ANPS) were completed by all participants; the Addiction Profile Index (API) was completed by the OUD patients to assess addiction‐related characteristics (addiction severity, motivation to quit substance use, craving, etc.).

***Sociodemographic data form*** is developed by the researchers. Sociodemographic and medical characteristics, including age, gender, marital status, education, employment status, financial comfort, chronic diseases, and mental disorders, were collected from all participants. In addition, information on the type of substance first used by OUD patients, the age at which they first started using substances, the duration of opioid use, and the route and daily dose of opioid use were obtained through the psychiatric interview and review of medical records.

***Affective Neuroscience Personality Scale*** developed by Davis et al. (2003) has been conspired as an adequate tool to evaluate primer affective systems and has used in many clinical studies (Davis et al., 2003). In this study, it was employed to evaluate participants’ affective personality traits. This 110‐item self‐report scale, graded on a 4‐point Likert scale, assesses six fundamental affect systems (PLAY, SEEK, CARE, FEAR, ANGER, SADNESS) alongside “Spirituality.” Each of these subscales comprises 14 questions, except for the Spirituality subscale, which contains 12 questions, all subscale questions evenly divided between positive and negative statements. The Turkish validity and reliability study was conducted by Özkarar‐Gradwohl et al. (2014).

***Addiction Profile Index*** was developed by Ögel et al. (2012) to assess problem dimensions related to alcohol and drug use, motivation to quit substance use, willingness to use substances intensively, and severity of dependence. This self‐report scale comprises 37 items distributed across five distinct subscales. These subscales evaluate patterns of substance use (PSU), diagnostic criteria for addiction (DIAG), the impact of substance use on the individual's life (ISU), craving (CRAV), and the motivation to quit substance use (MOTIV). Each subscale is scored on its own, and the score of each subscale is given equal weight in determining the total API score, that is, the severity of the addiction (Ögel et al., 2012).

### 2.1 Statistical analysis

All analyses were performed using IBM SPSS Statistics 24.0 (Statistical Package for the Social Sciences 24.0, Chicago, IL, USA). Descriptive statistics were presented for sociodemographic and clinical variables, as well as for scale scores. The Kolmogorov–Smirnov test and visual methods were used to check the distribution of continuous variables. The chi‐square test was used to compare categorical variables (gender, marital status, psychiatric and medical disorders, financial comfort, education, living arrangements, and military status). Mann–Whitney *U* test (ANPS) or student *t*‐test (API) was used for univariate analysis. Spearman's correlation coefficient, *ρ*, was used to evaluate the correlation between daily used heroin amount, age at first drug use, and scale scores. The possible factors identified with the univariate analysis were further entered into the stepwise logistic regression analysis to determine independent predictors of opiate use disorder and age at first drug use (before or after 17 years—the median age at first use of any substance was 17). Hosmer–Lemeshow goodness of fit statistics was used to assess model fit. All tests were two‐tailed, and a *p*‐value less than.05 was considered statistically significant.

## 3 RESULTS

In this study involving 141 opioid use disorder patients (OAP) and 160 healthy controls (HC), the mean age of all participants was 28.2 ± 6.8 years (min 18–max 51). Of the participants, 90.4% (*n* = 272) were male; 71% were single, divorced, or widowed; 69% (*n* = 208) had at least a high school education; and 47.5% (*n* = 143) had a full‐time or part‐time regular job.

The median (IQR) age at first use of any substance was 17 (6) years, the median (IQR) duration of opioid use was 6 (4.5) years, and the median (IQR) daily dose of heroin was 2 (2) g/day.

No differences were found between OAP and HC groups in terms of age, sex, marital status, employment status, and family psychiatric disorder history. It was found that 51 (36.2%) patients in the OAP group and 10 participants in the HC group had a family history of alcohol or drug use disorder (*p *< .001). Eighty‐seven healthy control participants did not answer the question about a family history of drug and alcohol use disorder.

When evaluating the ANPS subscales, a statistically significant difference was found between the OAP and HC groups for SEEK (*p* < .001), PLAY (*p* < .001), and SADNESS (*p* = .002). The comparison of sociodemographic characteristics and ANPS scores between the OAP and HC groups is presented in Table 1.

**TABLE 1:** Comparison of sociodemographic characteristics and scale scores between groups.

Characteristics	OAP (*n* = 141)	HC (*n* = 160)	Chi square/*z* *	*p*
Age, median (IQR)	28 (7)	26 (8.75)	−1.913*	.056
Gender, *n* (%)				
female	12 (8.5)	17 (10.6)	0.385	.535
male	129 (91.5)	143 (89.4)		
Marital status, *n* (%)				
Married/partnered	44 (31.2)	44 (27.5)	0.497	.481
Single/separate/widowed	97 (68.8)	116 (72.5)		
Employment status, *n* (%)				
Full or part‐time job	74 (52.5)	69 (43.1)	2.632	.105
Nonemployed/retired/ student/housewife	67 (47.5)	81 (56.9)		
Educational level, *n* (%)				
Literate and primary school	78 (55.3)	5 (3.3)	96.346	<.001
High school and above	63 (44.7)	145 (96.7)		
Family psychiatric history, *n* (%)				
Yes	21 (14.9)	25 (15.6)	0.031	.860
No	120 (85.1)	135 (84.4)		
Family alcohol and drug use disorder history, *n* (%)				
Yes	51 (36.2)	10 (12)	15.341	<.001
No	90 (63.8)	73 (88)		
ANPS, median (IQR)^a^				
SEEK	22 (5)	24 (6)	−4.333*	<.001
FEAR	22 (4)	22 (6)	−1.022*	.307
CARE	23 (9)	26 (7)	−1.909*	.056
ANGER	22 (5)	22 (7)	−0.443*	.608
PLAY	21 (5)	25 (7)	−5.063*	<.001
SADNESS	21 (4)	20 (6)	−3.051*	.002
Spirituality	18 (5)	20 (6)	−0.709*	.478
API, median (IQR)				
PSU	2.6 (2.6)			
DIAG	16 (6.6)			
ISU	31 (14.2)			
CRAV	10.5 (6)			
MOTIV	11 (3)			
API‐total	13 (4.2)			

IQR: interquartile range; OAP: opiate use disorder patients; HC: healthy controls; ANPS: the Affective Neuroscience Personality Scale; API‐Total: Addiction Profile Index Total Score; PSU: API patterns of substance use subscale; DIAG: API diagnostic criteria for addiction subscale; ISU: API the impact of substance use on the individual's life subscale; CRAV: API willingness to use substances intensively subscale; MOTIV: API motivation to quit substance use subscale.

^a^ Mann–Whitney *U* test.

^*^ *Z* score.

### 3.1 Correlation analysis

When examining the relationships between ANPS subscales and addiction severity (API‐Total) as well as motivation to quit addiction (MOTIV) in the OAP group, positive correlations were found between API‐Total and ANGER (*r* = .302, *p *< .01), and API‐Total and SADNESS (*r* = .209, *p *< .05). Additionally, we found negative correlation between API‐Total and PLAY (*r* = –.182, *p* < .05). Notably, no correlation was found between MOTIV and any of the ANPS subscales. Furthermore, a moderate negative correlation was observed between age at first drug use and ANGER (*r* = –.211, *p* < .05), as well as between age at first drug use and API‐Total (*r* = –.221, *p* < .01). On the other hand, positive correlations were noted between the daily dose of heroin and API‐Total (*r* = .458, *p* < .01), daily dose of heroin and CRAV (*r* = .456, *p* < .01), and daily dose of heroin and ANGER (*r* = .280, *p* < .01). Detailed correlation results are presented in Table 2.

**TABLE 2:** Correlations of scale scores of opioid use disorder group.

	Age at first drug use	Daily used heroin amount	PSU	DIAG	ISU	CRAV	MOTIV	API‐Total	SEEK	FEAR	CARE	ANGER	PLAY	SADNESS
**Daily used heroin amount**	–.323^**^													
**PSU**	–.161	.291^**^												
**DIAG**	–.106	.371^**^	.509^**^											
**ISU**	–.147	.334^**^	.384^**^	.704^**^										
**CRAV**	–.192^*^	.456^**^	.399^**^	.530^**^	.512^**^									
**MOTIV**	–.120	.213^*^	.000	.344^**^	.415^**^	.127								
**API‐Total**	–.221^**^	.458^**^	.608^**^	.860^**^	.832^**^	.720^**^	.479^**^							
**SEEK**	.019	–.018	–.175^*^	–.025	.022	–.114	.058	–.064						
**FEAR**	–.052	.020	.017	.104	.086	.093	.076	.097	.097					
**CARE**	.000	–.165	–.298^**^	–.107	–.132	–.208^*^	.263^**^	–.126	.374^**^	.119^*^				
**ANGER**	–.211^*^	.280^**^	.088	.231^**^	.269^**^	.245^**^	.161	.302^**^	.197^**^	.359^**^	.211^**^			
**PLAY**	.010	.022	–.198^*^	–.170^*^	–.130	–.192^*^	.110	–.182^*^	.463^**^	.068	.456^**^	.128^*^		
**SADNESS**	–.041	.151	.183^*^	.251^**^	.230^**^	.163	–.025	.209^*^	–.205^**^	.303^**^	–.098	.224^**^	–.184^**^	
**SPIRITUALITY**	–.112	–.096	–.144	–.061	–.071	–.215^*^	.110	–.106	.149^**^	.008	.442^**^	.130^*^	.224^**^	–.006

API‐Total: Addiction Profile Index Total Score; PSU: API patterns of substance use subscale, DIAG: API diagnostic criteria for addiction subscale; ISU: API the impact of substance use on the individual's life subscale; CRAV: API willingness to use substances intensively subscale; MOTIV: API motivation to quit substance use subscale.

^*^ *p* < .05.

^**^ *p* < .01.

### 3.2 Factors associated with opioid use disorder

We performed a logistic regression analysis to determine factors related to opioid use disorder. We added variables that demonstrated significant differences in paired comparisons and were previously reported in the literature as potential risk factors for opioid use disorder [family addiction history (no: 0, yes: 1), Education level (Literate and primary school: 0, High school and above: 1), and SEEK, CARE, ANGER, PLAY, SADNESS subscale scores of ANPS].

In the final model for opioid use disorder (Hosmer–Lemeshow test chi‐squared 6.844, *p* = .554), significant factors included a family history of alcohol or drug abuse, education, and PLAY subdomain of ANPS. The risk of opioid use disorder was higher in individuals with a family history of alcohol or drug abuse, low educational levels, and low PLAY affective personality traits (Table 3).

**TABLE 3:** Logistic regression analysis predicting opioid use disorder.

Predictor	Odds ratio (95% CI)	*p*
Family addiction history (yes)	4.933 (1.821–13.367)	.002
Education (High school and above)	.067 (0.024–0.184)	.000
SEEK	0.974 (0.886–1.070)	.582
CARE	1.056 (0.984–1.133)	.131
ANGER	1.007 (0.394–1.085)	.862
PLAY	0.902 (0.826–0.985)	.022
SADNESS	1.026 (0.947–1.111)	.528

*Note*: Nagelkerke *R* ^2^ = .418.

CI: confidence interval.

### 3.3 Factors associated with earlier onset of substance use

We performed a logistic regression analysis to determine factors related to earlier onset of substance use. Age at first drug use as a dependent variable (1: 17 years and younger, 0: after age 17). Factors examined were sex (male: 0, female: 1), family addiction history (no: 0, yes: 1), childhood trauma history (no: 0, yes: 1), PLAY and ANGER subscale scores. As outlined in Table 4, the presence of childhood trauma and high ANGER scores were identified as risk factors for earlier onset of substance use (Hosmer–Lemeshow test chi‐square 6.641, *p* = .576).

**TABLE 4:** Logistic regression analysis predicting to earlier onset of substance use.

Predictor	Odds ratio (95% CI)	*p*
Sex (female)	0.563 (0.160–1.980)	.371
Family addiction history (yes)	1.086 (0.517–2.282)	.827
Childhood trauma history (yes)	2.253 (1.091–4.650)	.028
PLAY	0.954 (0.878–1.035)	.258
ANGER	1.103 (1.023–1.190)	.011

*Note*: Nagelkerke *R* ^2^ = .119.

CI: confidence interval.

## 4 DISCUSSION

In this study, we aimed to identify the primary affective systems involved in opioid use disorder and determine the relationship between affective personality traits with motivational factors to quit opiate use, the severity of opioid addiction, and factors related to earlier onset of substance use.

In our study, we found differences in affective personality traits—specifically SEEK, PLAY, and SADNESS— between opioid use disorder patients and age‐ and sex‐matched healthy controls. Notably, in the regression model, family history of alcohol or drug abuse, low educational level, and low PLAY personality traits were identified as risk factors for opioid use disorder. Interestingly, we found no relationship between motivation to quit heroin use and any affective personality trait. However, there was a positive correlation between addiction severity and the SADNESS and ANGER affective personality traits. Although there are no studies in the literature examining affective personality traits in patients with nonmedical OUD, there are a limited number of studies in patients with SUDs.

In a study conducted by Unterrainer et al. (2017), individuals with multiple drug use disorders (Poly‐Drug Use, PUD), tobacco smokers (Recreational‐Drug Use, RUC), and nonsmokers who stated that they had never used illicit substances (Non‐Drug Users, NUC) were all given the APNS. When the PUD group was compared with the other two groups, higher scores were found for ANGER, FEAR, and SADNESS, but no significant differences were found for the SEEK, CARE, and PLAY scales. In addition, the RUC (*p* < .05) and PUD (*p* < .01) groups had higher ANGER scores, and only the PUD group had higher SADNESS and FEAR scores when compared with normative data from the general population (Unterrainer et al., 2017).

The difference in SEEK scores in our study may be related to SUD‐related clinical symptoms such as craving phenomenology. SEEKING is a dopamine‐driven system, and its hyperactivation has been reported to be associated with substance use disorder due to its relationship with the reward system (Alcaro & Panksepp, 2011; Wright & Panksepp, 2012). The fact that SEEK scores were not high in Unterrainer's study has been interpreted as the possibility that the high SADNESS scores of the participants may have silenced the SEEK circuit (Unterrainer et al., 2017). We found high scores of SADNESS and SEEK in our study. In another study on a sample of psychiatric patients, the dimensions of SADNESS and ANGER were found to play a pivotal role in substance abuse by Fuchshuber et al. (2019).

Exogenous opioid agonists suppress avoidance‐oriented feelings, such as fear and sadness, while promoting approach‐oriented feelings, such as anger and pleasure. In addition, opioids affect affiliative behavior and social bonding (Inagaki et al., 2020; Nummenmaa et al., 2016). The PLAY primary affective system is crucial for fostering social bonds, developing social skills, developing motor competence, and possibly contributing to neocortical emotion control (Montag et al., 2021). PLAY is also crucial in depressive tendencies, together with SEEK, ANGER, and FEAR (Fuchshuber et al., 2019; Montag & Panksepp, 2017). In the literature, negative mood states consistently correlate with more severe opioid use disorder (Martel et al., 2014; McCabe et al., 2007; Merlo et al., 2013). In university students, using opiates to manage negative mood is associated with more severe opioid use disorder (McCabe et al., 2007; Merlo et al., 2013). Moreover, there exists a correlation between escalated nonmedical opioid use and negative affectivity, a relatively stable and heritable tendency to experience nonspecific distress or unpleasant emotions (Edwards et al., 2016; Martel et al., 2014; Wasan et al., 2015). Recent research suggests that opioids engage the brain's stress and pain (emotional and somatic) systems and result in hyperalgesia and hyperkatifeia (a hypersensitive negative emotional state associated with drug withdrawal)—which, via negative reinforcement mechanisms, drive significant drug‐seeking behavior (Koob, 2020). Through opioid craving, there is an indirect association between negative affectivity and an elevated risk of nonmedical opioid use (Martel et al., 2014). In their study, Bakhshaie et al. (2019) stated that individuals with high negative affect, irrespective of pain experience, may be prone to opioid‐related issues, partly due to emotional dysregulation. Considering all these data, our findings that low PLAY affective personality traits pose a risk for opioid use disorder and the positive correlation between addiction severity and the SADNESS and ANGER affective personality traits are consistent with the literature.

Our study found a significant difference in educational levels between OUD patients and the HC group. Although this difference in education levels among the groups seems to be a confounding factor, the result is consistent with the literature. The relationship between substance addiction and education is multifaceted. One study reported that adolescents with past‐year substance use are more likely to skip school and have lower grade averages, even after adjusting for demographic variables (Bugbee et al., 2019). Studies have reported that a low level of education can increase the risk of substance use, while substance use can, in turn, negatively impact educational success and cognitive performance (Bugbee et al., 2019; Melugin et al., 2021). However, this relationship is also influenced by various external factors, including cultural, economic, and social factors. Further research is needed to explore the relationship between education levels and opioid use.

Similar to the literature, we found that a family history of substance use has been associated with opioid addiction risk (Prasant et al., 2006). Demetrovics (2000) indicated that the roots of opiate addiction can be found in early life. Although both psychological and physiological mechanisms influence the development of endogenous opioid systems, this view also supports the influence of primary affective systems, which are evolutionarily passed down and interact to shape the cortex.

Furthermore, we found a relationship between age at first drug use and the high amount of heroin used per day and ANGER. In addition, the presence of childhood trauma and high ANGER scores were identified as risk factors for earlier onset of substance use. The ANGER subscale is defined by irritation, frustration, and outward and verbal expressions of anger (Brienza et al., 2023). The association of the ANGER primary affective system with irritability is consistent in the literature. Irritability increases the risk of depression and anxiety, which in turn increases the risk of substance abuse (Vidal‐Ribas et al., 2016). In addition, childhood‐onset irritability is associated with substance use in early adulthood as a maladaptive way of coping with anger (Bilaç et al., 2021; Tarter et al., 1995). In studies of patients with SUD, irritability is particularly associated with opioid use (Antón‐Galindo et al., 2023; Rovai et al., 2017). It has been reported in a recent meta‐analysis that people who use psychoactive substances have higher anger trait scores than nonusers, and the relapse rate after treatment is higher for patients with high anger scores (Laitano et al., 2022). The relationship between opioid use and anger has also led to the hypothesis that opioid use disorder patients may attempt to self‐medicate to cope with anger (Aharonovich et al., 2001). These findings suggest that regulation of the ANGER primary affective system will be important in preventing substance use as a way of coping at an early age and in the treatment of opioid use disorder.

Our study has several limitations. First, the cross‐sectional design may have limited our ability to determine causality. Furthermore, convenience sampling, the single‐center design, and the small number of female patients may have increased the risk of a nonrepresentative sample and limited generalizability. Although psychiatrists carried out the assessments to determine active psychopathology, the lack of objective methods (e.g., SCID‐5) to assess other psychiatric disorders related to substance use and the failure to record the psychotropic drugs used by patients are additional limitations. Although the scale questions were used to assess the level of motivation to quit opioid use, the lack of distinction between patients who voluntarily applied for treatment and those referred by the court may explain the lack of relationship between motivational factors and affective personality traits. Finally, the absence of a family history of SUDs in nearly half of the HC group may restrict the conclusions that can be drawn about the relationship between a family history of SUDs and affective personality traits. Despite these limitations, our study has many clinical implications.

In conclusion, this study is the first to examine the relationship between affective neuroscience personality traits, opioid addiction severity, earlier onset of substance use, and motivation to quit opioid use. We identified low PLAY personality trait as a risk factor for opioid use disorder, whereas childhood trauma and high ANGER personality trait were risk factors for earlier onset of substance use. Our findings underscore the multifaceted nature of vulnerability to SUDs. The identification of these affective systems may have implications for the development of personalized prevention and treatment strategies. Individuals with a high ANGER trait may benefit from tailored anger management therapies or specific psychotropic medications aimed at modulating affective dysregulation. Similarly, recognizing low PLAY as a risk factor could guide the development of interventions that enhance positive affective experiences and community‐based activities to stimulate social engagement and pleasure. Additionally, the early identification of these traits in at‐risk populations may offer opportunities for prevention, such as targeted psychosocial interventions in adolescence to mitigate the development of substance use disorders.

## AUTHOR CONTRIBUTIONS

**Gonca Aşut**: Conceptualization; funding acquisition; writing—original draft; writing—review and editing; resources; formal analysis; methodology; visualization. **Yasemin Hoşgören Alıcı**: Supervision; resources; writing—review and editing; methodology; conceptualization. **Selvi Ceran**: Conceptualization; writing—review and editing; supervision; resources; formal analysis. **Mustafa Danışman**: Conceptualization; writing—review and editing; resources; data curation. **Şafak Yalçın Şahiner**: Data curation; resources; supervision; writing—review and editing; conceptualization.

## CONFLICT OF INTEREST STATEMENT

The authors declare no potential conflict of interest.

### PEER REVIEW

The peer review history for this article is available at https://publons.com/publon/10.1002/brb3.70050.

## CONSENT TO PARTICIPATE STATEMENT

Written informed consent was obtained from all participants.

## Data Availability

The datasets used and analyzed during the current study are available from the corresponding author upon reasonable request.

## References

[brb370050-bib-0001] Aharonovich, E. , Nguyen, H. T. , & Nunes, E. V. (2001). Anger and depressive states among treatment‐seeking drug abusers: Testing the psychopharmacological specificity hypothesis. American Journal on Addictions, 10(4), 327–334. 11783747

[brb370050-bib-0002] Alcaro, A. , & Panksepp, J. (2011). The SEEKING mind: Primal neuro‐affective substrates for appetitive incentive states and their pathological dynamics in addictions and depression. Neuroscience & Biobehavioral Reviews, 35(9), 1805–1820. 21396397 10.1016/j.neubiorev.2011.03.002

[brb370050-bib-0003] Antón‐Galindo, E. , Cabana‐Domínguez, J. , Torrico, B. , Corominas, R. , Cormand, B. , & Fernàndez‐Castillo, N. (2023). The pleiotropic contribution of genes in dopaminergic and serotonergic pathways to addiction and related behavioral traits. Frontiers in Psychiatry, 14, 1293663. 10.3389/fpsyt.2023.1293663. 37937232 PMC10627163

[brb370050-bib-0004] Bakhshaie, J. , Rogers, A. H. , Kauffman, B. Y. , Tran, N. , Buckner, J. D. , Ditre, J. W. , & Zvolensky, M. J. (2019). Emotion dysregulation as an explanatory factor in the relation between negative affectivity and non‐medical use of opioid in a diverse young adult sample. Addictive Behaviors, 95, 103–109. 10.1016/j.addbeh.2019.02.025. 30877901 PMC7061416

[brb370050-bib-0005] Bilaç, Ö. , Önder, A. , Kavurma, C. , Uzunoğu, G. , & Sapmaz, Ş. (2021). Maternal attitudes, irritability, problem solving skills and decision making in adolescents with substance use: Case‐control study. Turk Psikiyatri Dergisi = Turkish Journal of Psychiatry, 32(1). 10.5080/u25055 34181740

[brb370050-bib-0006] Brienza, L. , Zennaro, A. , Vitolo, E. , & Andò, A. (2023). Affective Neuroscience Personality Scale (ANPS) and clinical implications: A systematic review. Journal of Affective Disorders, 320, 178–195. 10.1016/j.jad.2022.09.104. 36174784

[brb370050-bib-0007] Bugbee, B. A. , Beck, K. H. , Fryer, C. S. , & Arria, A. M. (2019). Substance use, academic performance, and academic engagement among high school seniors. Journal of School Health, 89(2), 145–156. 10.1111/josh.12723. 30604451 PMC6373775

[brb370050-bib-0008] Clark, R. E. , Baxter, J. D. , Aweh, G. , O'Connell, E. , Fisher, W. H. , & Barton, B. A. (2015). Risk factors for relapse and higher costs among Medicaid members with opioid dependence or abuse: Opioid agonists, comorbidities, and treatment history. Journal of Substance Abuse Treatment, 57, 75–80. 10.1016/j.jsat.2015.05.001. 25997674 PMC4560989

[brb370050-bib-0009] Davis, K. L. , Panksepp, J. , & Normansell, L. (2003). The affective neuroscience personality scales: Normative data and implications. Neuropsychoanalysis, 5(1), 57–69. 10.1080/15294145.2003.10773410.

[brb370050-bib-0010] Demetrovics, Z. (2000). Family‐history perspective of opiate addiction. Focusing on pre‐and perinatal events. International Journal of Prenatal and Perinatal Psychology and Medicine, 12(3), 443–466.

[brb370050-bib-0011] Dydyk, A. , Jain, N. , & Gupta, M. (2023). Opioid use disorder [Updated 2023 Jul 21]. StatPearls Publishing. 31985959

[brb370050-bib-0012] Edwards, R. , Dolman, A. , Michna, E. , Katz, J. , Nedeljkovic, S. , Janfaza, D. , Isaac, Z. , Martel, M. O. , Jamison, R. N. , & Wasan, A. (2016). Changes in pain sensitivity and pain modulation during oral opioid treatment: The impact of negative affect. Pain Medicine, 17(10), 1882–1891. 10.1093/pm/pnw010. 26933094 PMC5063023

[brb370050-bib-0013] Flores Mosri, D. (2019). Affective features underlying depression in addiction: Understanding what it feels like. Frontiers in Psychology, 10, 2318. 10.3389/fpsyg.2019.02318. 31681110 PMC6811663

[brb370050-bib-0014] Flores Mosri, D. (2021). Affective neuroscience contributions to the treatment of addiction: The role of social instincts, pleasure and SEEKING. Frontiers in Psychiatry, 2096. 10.3389/fpsyt.2021.761744 PMC8649919 34887789

[brb370050-bib-0015] Fuchshuber, J. , Hiebler‐Ragger, M. , Kresse, A. , Kapfhammer, H.‐P. , & Unterrainer, H. F. (2018). Depressive symptoms and addictive behaviors in young adults after childhood trauma: The mediating role of personality organization and despair. Frontiers in Psychiatry, 9, 318. 10.3389/fpsyt.2018.00318. 30061848 PMC6054985

[brb370050-bib-0016] Fuchshuber, J. , Hiebler‐Ragger, M. , Kresse, A. , Kapfhammer, H.‐P. , & Unterrainer, H. F. (2019). The influence of attachment styles and personality organization on emotional functioning after childhood trauma. Frontiers in Psychiatry, 10, 643. 10.3389/fpsyt.2019.00643. 31543844 PMC6739441

[brb370050-bib-0017] Inagaki, T. K. , Hazlett, L. I. , & Andreescu, C. (2020). Opioids and social bonding: Effect of naltrexone on feelings of social connection and ventral striatum activity to close others. Journal of Experimental Psychology: General, 149(4), 732. 10.1037/xge0000674. 31414860 PMC7021584

[brb370050-bib-0018] Koob, G. F. (2020). Neurobiology of opioid addiction: Opponent process, hyperkatifeia, and negative reinforcement. Biological Psychiatry, 87(1), 44–53. 10.1016/j.biopsych.2019.05.023. 31400808

[brb370050-bib-0019] Laitano, H. V. , Ely, A. , Sordi, A. O. , Schuch, F. B. , Pechansky, F. , Hartmann, T. , Hilgert, J. B. , Wendland, E. M. , Von Dimen, L. , Scherer, J. N. , Calixto, A. M. , Narvaez, J. C. M. , Ornell, F. , & Kessler, F. H. P. (2022). Anger and substance abuse: A systematic review and meta‐analysis. Brazilian Journal of Psychiatry, 44, 103–110. 10.1590/1516-4446-2020-1133. 33605366 PMC8827371

[brb370050-bib-0020] Majewska, M. D. (1996). Cocaine addiction as a neurological disorder: Implications for treatment. NIDA Research Monograph, 163, 1–26. 8809851

[brb370050-bib-0021] Martel, M. O. , Dolman, A. J. , Edwards, R. R. , Jamison, R. N. , & Wasan, A. D. (2014). The association between negative affect and prescription opioid misuse in patients with chronic pain: The mediating role of opioid craving. The Journal of Pain, 15(1), 90–100. 10.1016/j.jpain.2013.09.014. 24295876 PMC3877217

[brb370050-bib-0022] McCabe, S. E. , Cranford, J. A. , Boyd, C. J. , & Teter, C. J. (2007). Motives, diversion and routes of administration associated with nonmedical use of prescription opioids. Addictive Behaviors, 32(3), 562–575. 10.1016/j.addbeh.2006.05.022. 16843611 PMC1766373

[brb370050-bib-0023] Melugin, P. R. , Nolan, S. O. , & Siciliano, C. A. (2021). Bidirectional causality between addiction and cognitive deficits. International Review of Neurobiology, 157, 371–407. 10.1016/bs.irn.2020.11.001. 33648674 PMC8566632

[brb370050-bib-0024] Merlo, L. J. , Singhakant, S. , Cummings, S. M. , & Cottler, L. B. (2013). Reasons for misuse of prescription medication among physicians undergoing monitoring by a physician health program. Journal of Addiction Medicine, 7(5), 349–353. 10.1097/ADM.0b013e31829da074. 24089039 PMC3790148

[brb370050-bib-0025] Montag, C. , Elhai, J. D. , & Davis, K. L. (2021). A comprehensive review of studies using the Affective Neuroscience Personality Scales in the psychological and psychiatric sciences. Neuroscience & Biobehavioral Reviews, 125, 160–167. 33609568 10.1016/j.neubiorev.2021.02.019

[brb370050-bib-0026] Montag, C. , & Panksepp, J. (2016). Primal emotional‐affective expressive foundations of human facial expression. Motivation and Emotion, 40, 760–766. 10.1007/s11031-016-9570-x.

[brb370050-bib-0027] Montag, C. , & Panksepp, J. (2017). Primary emotional systems and personality: An evolutionary perspective. Frontiers in Psychology, 8, 253148. 10.3389/fpsyg.2017.00464 PMC5387097 28443039

[brb370050-bib-0028] Nummenmaa, L. , Tuominen, L. , Dunbar, R. , Hirvonen, J. , Manninen, S. , Arponen, E. , Machin, A. , Hari, R. , Jääskeläinen, I. P. , & Sams, M. (2016). Social touch modulates endogenous μ‐opioid system activity in humans. Neuroimage, 138, 242–247. 10.1016/j.neuroimage.2016.05.063. 27238727

[brb370050-bib-0029] Ögel, K. , Evren, C. , Karadağ, F. , & Gürol, T. (2012). Bağımlılık Profil İndeksi'nin (BAPİ) geliştirilmesi, geçerlik ve güvenilirliği. Turk Psikiyatri Dergisi = Turkish Journal of Psychiatry, 23(4), 264–273. 23225127

[brb370050-bib-0030] Özkarar‐Gradwohl, F. , Panksepp, J. , İçöz, F. , Çetinkaya, H. , Köksal, F. , Davis, K. , & Scherler, N. (2014). The influence of culture on basic affective systems: The comparison of Turkish and American norms on the affective neuroscience personality scales. Culture and Brain, 2, 173–192. 10.1007/s40167-014-0021-9.

[brb370050-bib-0031] Panksepp, J. (2004). Affective neuroscience: The foundations of human and animal emotions. Oxford University Press.

[brb370050-bib-0032] Panksepp, J. , & Biven, L. (2012). The archaeology of mind: Neural origins of human emotion. WW Norton & Company.

[brb370050-bib-0033] Prasant, M. P. , Mattoo, S. K. , & Basu, D. (2006). Substance use and other psychiatric disorders in first‐degree relatives of opioid‐dependent males: A case–controlled study from India. Addiction, 101(3), 413–419. 10.1111/j.1360-0443.2006.01340.x. 16499514

[brb370050-bib-0034] Rovai, L. , Maremmani, A. G. , Bacciardi, S. , Gazzarrini, D. , Pallucchini, A. , Spera, V. , Perugi, G. , & Maremmani, I. (2017). Opposed effects of hyperthymic and cyclothymic temperament in substance use disorder (heroin‐or alcohol‐dependent patients). Journal of Affective Disorders, 218, 339–345. 10.1016/j.jad.2017.04.041. 28494392

[brb370050-bib-0035] Tarter, R. E. , Blackson, T. , Brigham, J. , Moss, H. , & Caprara, G. V. (1995). The association between childhood irritability and liability to substance use in early adolescence: A 2‐year follow‐up study of boys at risk for substance abuse. Drug and Alcohol Dependence, 39(3), 253–261. 10.1016/0376-8716(95)01175-6. 8556975

[brb370050-bib-0036] Unterrainer, H.‐F. , Hiebler‐Ragger, M. , Koschutnig, K. , Fuchshuber, J. , Tscheschner, S. , Url, M. , Wagner‐Skacel, J. , Reininghaus, E. Z. , Papousek, I. , Weiss, E. M. , & Fink, A. (2017). Addiction as an attachment disorder: White matter impairment is linked to increased negative affective states in poly‐drug use. Frontiers in Human Neuroscience, 11, 208. 10.3389/fnhum.2017.00208. 28503141 PMC5408064

[brb370050-bib-0037] Vidal‐Ribas, P. , Brotman, M. A. , Valdivieso, I. , Leibenluft, E. , & Stringaris, A. (2016). The status of irritability in psychiatry: A conceptual and quantitative review. Journal of the American Academy of Child & Adolescent Psychiatry, 55(7), 556–570. 27343883 10.1016/j.jaac.2016.04.014 PMC4927461

[brb370050-bib-0038] Wasan, A. D. , Michna, E. , Edwards, R. R. , Katz, J. N. , Nedeljkovic, S. S. , Dolman, A. J. , Janfaza, D. , Isaac, Z. , & Jamison, R. N. (2015). Psychiatric comorbidity is associated prospectively with diminished opioid analgesia and increased opioid misuse in patients with chronic low back pain. Anesthesiology, 123(4), 861–872. 10.1097/ALN.0000000000000768. 26375824 PMC4573512

[brb370050-bib-0039] Wright, J. S. , & Panksepp, J. (2012). An evolutionary framework to understand foraging, wanting, and desire: The neuropsychology of the SEEKING system. Neuropsychoanalysis, 14(1), 5–39. 10.1080/15294145.2012.10773683.

